# Islet cells in human type 1 diabetes: from recent advances to novel therapies – a symposium-based roadmap for future research

**DOI:** 10.1530/JOE-23-0082

**Published:** 2023-08-31

**Authors:** J Cantley, D L Eizirik, E Latres, C M Dayan, the JDRF-DiabetesUK-INNODIA-nPOD Stockholm Symposium 2022

**Affiliations:** 1 School of Medicine, University of Dundee, Dundee, United Kingdom of Great Britain and Northern Ireland; 2 ULB Center for Diabetes Research, Université Libre de Bruxelles Faculté de Médecine, Bruxelles, Belgium; 3 JDRF International, New York, NY, USA; 4 Cardiff University School of Medicine, Cardiff, United Kingdom of Great Britain and Northern Ireland

**Keywords:** islet, pancreas, insulin secretion, autoimmune, diabetes

## Abstract

There is a growing understanding that the early phases of type 1 diabetes (T1D) are characterised by a deleterious dialogue between the pancreatic beta cells and the immune system. This, combined with the urgent need to better translate this growing knowledge into novel therapies, provided the background for the JDRF–DiabetesUK–INNODIA–nPOD symposium entitled ‘Islet cells in human T1D: from recent advances to novel therapies’, which took place in Stockholm, Sweden, in September 2022. We provide in this article an overview of the main themes addressed in the symposium, pointing to both promising conclusions and key unmet needs that remain to be addressed in order to achieve better approaches to prevent or reverse T1D.

## Introduction

**Figure 1:**
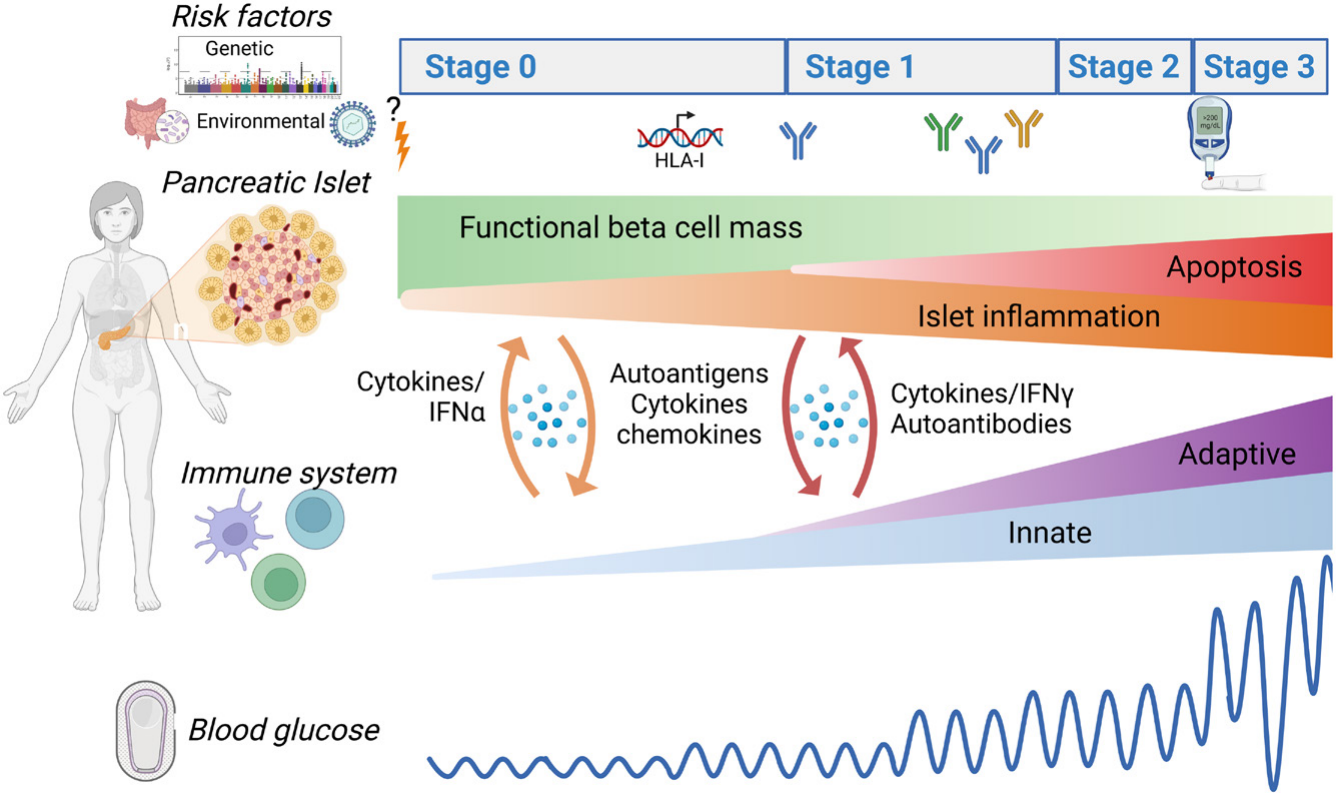
Progression to type 1 diabetes (T1D). Risk factors for T1D include genetic predisposition and environmental factors, such as viral infections and diet. Although the clinical diagnosis of T1D usually occurs at stage 3 when beta cell mass is depleted and hyperglycaemia is established, several stages precede this. There is increasing recognition of stage 0, in which beta cell dysfunction and impaired glycaemic control may occur in the absence of autoantibodies. Stage 1 represents seroconversion with the engagement of the adaptive immune system and the appearance of autoantibodies. During stage 2 beta cell dysfunction and apoptosis are increased impairing glycaemic control, leading to clinical diagnosis at stage 3. An important concept discussed is the reciprocal interaction between the beta cell and immune system throughout disease progression, along with interperson heterogeneity influenced by factors such as age of onset. Figure created with BioRender.com.

Type 1 diabetes (T1D) is a chronic autoimmune disease with a strong inflammatory component that results from the interaction between predisposing genes and environmental factors (e.g. viral infections and diet). This interaction triggers an autoimmune assault against pancreatic beta cells that provokes local inflammation (insulitis) and progressive beta cell functional impairment as well as the loss due to apoptosis. The prevalence of T1D has doubled in 25 years in children, and people living with the disease lose on average ≥11 years of life expectancy (Livingstone *et al.* 2015, Gregory *et al.* 2022). Although interventions targeting the immune system have shown efficacy in delaying disease onset (Herold *et al.* 2019), there are presently no therapies that prevent or cure the disease. The limited success of drugs aiming to arrest/revert autoimmunity and to protect beta cells in T1D is mainly due to the poor understanding of the molecular pathogenesis driving the autoimmune destruction of human beta cells, as well as the heterogeneity of pathogenesis resulting in different patient ‘endotypes’ (Bluestone *et al.* 2021, Eizirik *et al.* 2021). Immune-mediated inflammation (‘initiation phase’) contributes both to the primary induction and secondary amplification of the immune assault, and inflammatory mediators trigger functional suppression and apoptosis of beta cells. This takes place in the context of a ‘dialogue’ between invading immune cells and the target beta cells, which is modulated by T1D candidate genes acting both on the immune system and on the beta cells (Polychronakos & Li 2011, Bergholdt *et al.* 2012, Eizirik *et al.* 2012, Eizirik *et al.* 2020). This dialogue is mediated by cytokines/chemokines released by beta cells, immune cells and by putative immunogenic signals delivered by dying or ‘altered’ beta cells. Immune cells – particularly CD8^+^ T cells that recognise antigens presented in the context of conventional human leukocyte antigen class I (HLA-I) – mediate beta cell apoptosis. Beta cells contribute to this process by upregulating the expression of the HLA-I molecules. The upregulated HLA-I may present neoantigens generated as a consequence of beta cell endoplasmic reticulum (ER) stress, posttranslational modifications, changes in alternative splicing (AS) and/or other mechanisms (Gonzalez-Duque *et al.* 2018, Azoury *et al.* 2020, Piganelli *et al.* 2020, Roep *et al.* 2021). The pathogenesis of T1D has been proposed to consist of stages 1–3, preceded by a putative stage 0 (prior to seroconversion), as depicted in Fig. 1. It is important to state that not all individuals at stage 1 (autoantibody positive) will progress to stage 3 (hyperglycaemia and clinical diagnosis), and there is heterogeneity in disease progression and presentation.

The rationale for the JDRF–DiabetesUK–INNODIA–nPOD symposium entitled ‘Islet cells in human T1D: from recent advances to novel therapies’, which took place in Stockholm, Sweden, between September 23 and 25, 2022, was provided by the following observations: (i) the key role played by the deleterious dialogue between the immune system and the pancreatic beta cells in triggering the autoimmune process in T1D (Fig. 1); (ii) the urgent need to better translate this growing knowledge into novel (and probably combined) therapies for the disease, including better alignment of preclinical research with these goals. The main points discussed in the symposium are outlined later, culminating in a series of conclusions that should hopefully serve as a roadmap for future research in the field, with the ultimate goal to prevent the progressive beta cell dysfunction and loss characteristic of the disease.

## The progressive beta cell immune system dialogue in T1D and functional follow-up in clinical trials

The meeting started with an overview lecture provided by Chantal Mathieu, emphasising the importance of the dialogue between beta cells and the immune system and the role of chemokines in this process. The understanding that beta cells are not simply inert victims of the immune system, but contribute to the natural history of the disease (Martens *et al.* 2021), is promoting the search for combined therapies to prevent or revert T1D, aiming to both ‘re-educate’ the immune system and boost the beta cell’s resistance to the immune assault. This provides the basis for novel and planned clinical trials implemented by the European consortium INNODIA (https://www.innodia.eu/).

### Human islets in health and disease: the phenotype of human pancreatic beta cells in T1D

**Figure 2:**
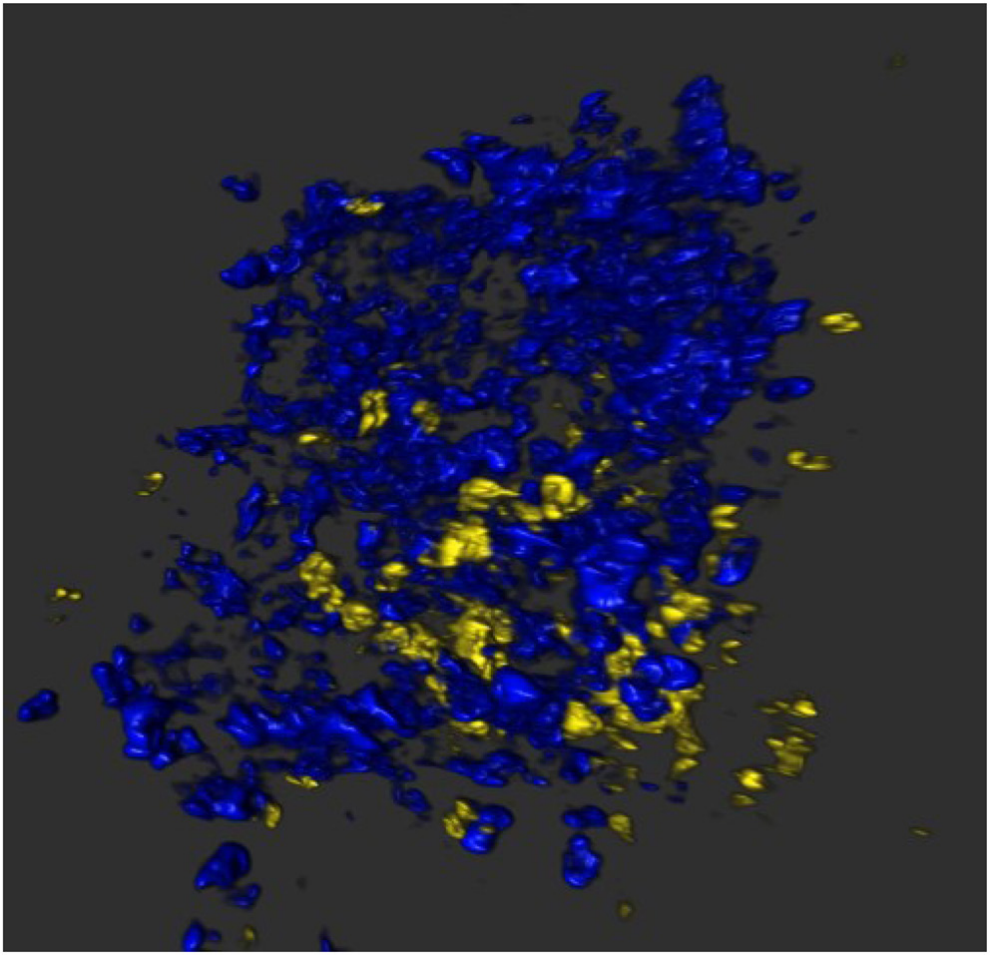
Visualising insulitis in three dimensions. Image of a 3D reconstruction of an nPOD donor (20-year-old T1D subject with 7 months disease duration) stained for glucagon (blue) and CD3 (yellow), revealing the presence of infiltrating immune cells within the islet of Langerhans, interspersed with endocrine cells. 3D reconstruction was performed using Arivis Vision4D.

Mark Atkinson presented a lecture titled, ‘The Network for Pancreatic Organ Donors with Diabetes (nPOD) experience: histology of beta cells in T1D’ where he discussed the impact that improvements to existing technologies (e.g. confocal microscopy and light sheet fluorescent microscopy), new image analysis methods and the development of novel technologies (e.g. Co-Detection by Imaging (CODEX), Cytometry by Time of Flight (CyTOF)) have made on our mechanistic understanding of T1D pathogenesis. These technologies, combined with the availability of extremely rare and valuable tissues from individuals with or at increased risk of T1D, have led to many discoveries that might be considered ‘transformational’ in terms of their thought. Indeed, these recent advancements have uncovered multiple previously undescribed physiological contributors to T1D, many related to aspects of not only the endocrine but extending to the exocrine pancreas. To be clear, evidence for the autoimmune component in T1D remains strong, including the presentation of a moving 3D image of an nPOD donor (20-year-old T1D subject with 7 months disease duration) stained for glucagon and CD3, clearly demonstrating the presence of immune cells within the islet, interspersed amongst endocrine cells (Fig. 2).

Klaus Kaestner discussed the ‘Transcriptomics of beta cells from T1D pancreata’ and the role played by the Human Pancreas Analysis Programme (HPAP), a consortium undertaking deep phenotyping of the human endocrine pancreas and its interaction with the immune system. HPAP generates and distributes high-value data sets for the diabetes research community (Kaestner *et al.* 2019). Pancreatic and immune tissues undergo histology, multi-omics and cellular studies before data are integrated into PancDB (https://hpap.pmacs.upenn.edu/). The programme has made a rapid impact in the field (Wang *et al.* 2019, Fasolino *et al.* 2020, Japp *et al.* 2021, Bosi *et al.* 2022, Doliba *et al.* 2022, Fasolino *et al.* 2022). HPAP single-cell multi-omics is enabling new insights into pancreatic subpopulations in the T1D pancreas. For example, these data enabled the recent identification of major histocompatibility complex (MHC)-class II transcripts in a novel subset of pancreatic ductal epithelial cells during T1D, with a tolerogenic dendritic cell gene expression profile suggesting attempted immune regulation (Fasolino *et al.* 2022). Dr Kaestner went on to discuss alpha cell hypersecretion of glucagon from islets in non-diabetic glutamic acid decarboxylase (GAD) autoantibody-positive individuals: single-cell RNAseq (scRNAseq) has identified upregulation of alpha cell glycolytic genes, which may underpin the augmented glucagon release in the GADA+ pancreas (Doliba *et al.* 2022), consistent with other mechanistic studies by the group (Bahl *et al.* 2021). The lecture concluded with a recent transcriptional analysis of residual beta cells in the T1D pancreas (Wang *et al.* 2019).

Marcela Brissova considered the role of ‘islet cell physiology in T1D’, a disease of dysregulated glucose homeostasis. Currently, all forms of diabetes are diagnosed by elevated blood glucose. The failure of clinical diagnosis and treatment to incorporate molecular or genetic features of diabetes is a limiting factor in building efforts towards precision medicine and disease prevention. To bridge this gap, several resources in the USA are generating multimodal data from the human pancreas or islets supported by robust organ donation mechanisms. These resources include HPAP (Kaestner *et al.* 2019) (https://hpap.pmacs.upenn.edu/), nPOD (https://www.jdrfnpod.org), Human Islet Phenotyping Program of IIDP (Brissova *et al.* 2019) (https://iidp.coh.org), Human BioMolecular Atlas Program (2019) (https://hubmapconsortium.org) and the Human Atlas of the Neonatal and Early Life Pancreas (https://pancreatlas.org/datasets/531/overview). Dr Brissova and her colleagues at Vanderbilt University participate on various levels in all five programs, four of which are currently connected via the data-agnostic Pancreatlas platform (Saunders *et al.* 2020) (https://pancreatlas.org/), a curated resource to share imaging datasets developed by the Vanderbilt team. To integrate functional, genomic and genetic data with key donor characteristics across age, sex, ancestry and disease state, a robust islet phenotyping pipeline was developed (Brissova *et al.* 2018, Hart *et al.* 2018, Haliyur *et al.* 2019, Haliyur *et al.* 2021, Doliba *et al.* 2022) that allowed analyses of human islet preparations from >600 donors thus far including adult and paediatric individuals with and without T1D (Brissova *et al.* 2018, Haliyur *et al.* 2021). These approaches revealed that endocrine cell composition varies considerably in adult islets and is highly associated with hormone secretion, possibly influencing alpha–beta–delta cell paracrine interactions. In addition, the team found that beta cell composition and insulin secretion are influenced by sex and self-reported ancestry. During postnatal development, islet endocrine cell composition changes considerably and both beta and alpha cells undergo functional maturation, thus further raising questions about gene(s) and environmental factors influencing endocrine cell allocation, beta cell mass and diabetes predisposition. Studies of isolated islets from individuals diagnosed with T1D (stage 3) showed that residual beta cells have relatively normal function, whereas alpha cells have an intrinsic defect resulting in dysregulated glucagon secretion. Brissova postulated that several mechanisms could be responsible for alpha cell dysfunction in T1D such as extreme beta cell loss, chronic hyperglycaemia or repeated hypoglycaemia leading to changes in alpha cell state mediated by alterations in the expression of key islet-enriched transcription factors (TFs) (Brissova *et al.* 2018, Wang *et al.* 2019, Camunas-Soler *et al.* 2020). Recent work using islets from non-diabetic adult single GAD autoantibody-positive individuals led to a surprising discovery that alpha cells appear to have dysregulated secretory output prior to symptomatic T1D (Doliba *et al.* 2022). Collectively, these insights demonstrate that T1D is a complex disease requiring integrated studies by collaborative interdisciplinary teams to bring molecular insight into heterogeneity across multiple levels to achieve precision medicine in diagnosis and management. Integrating cellular function with molecular signatures and genetics, whilst fostering interactions of basic scientists and clinicians could inform future clinical trials aiming at T1D prevention.

‘What do the islets look like in T1D?’ To address this question, Sarah Richardson reflected on the value of pancreas biobanks in which T1D islets can be examined in their native environment. Globally there are <700 T1D donor pancreata, with the majority accessible today residing within the nPOD and the Exeter Archival Diabetes Biobanks (EADB; https://pancreatlas.org/datasets/960/explore). Advanced technologies such as scRNAseq and CyToF are providing exciting and informative insights into the pathology of T1D (Damond *et al.* 2019, Wang *et al.* 2019) but are frequently restricted to a small subset of individuals diagnosed between 10 and 30 years of age, with a duration of diabetes of >2 years. Donors with young-onset (age <10 years) and short duration (<2 years); those with long duration (>35 years); and those with late-onset (age >30 years) receive less attention due to limited access. The need to study ‘all’ groups is highlighted by the variation in hallmark T1D-related pathologies, which differ depending on ‘who you look in’ and ‘when you look’.

**Figure 3:**
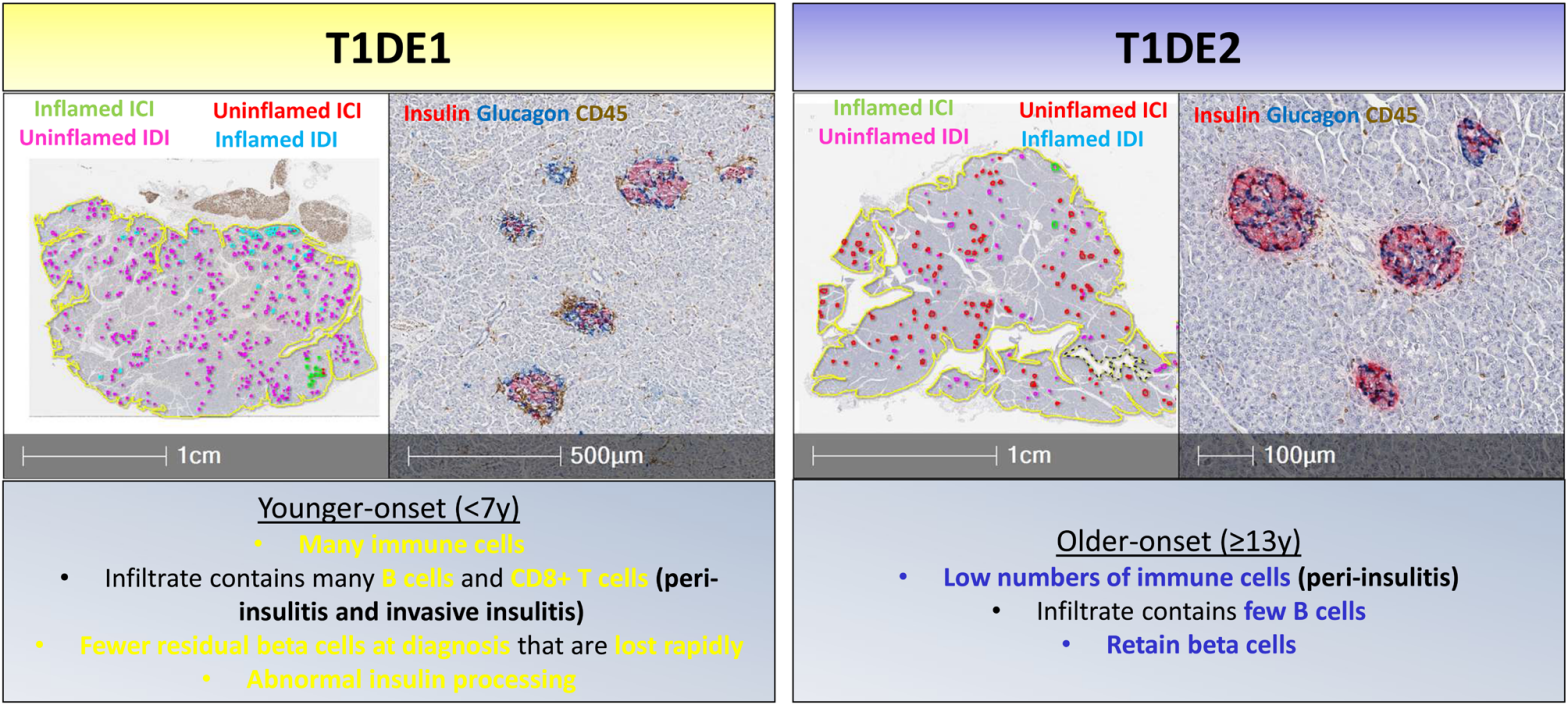
Summary of pancreas Type 1 diabetes endotypes (T1DE). Images of pancreatic sections from a T1DE1 (left panel) or T1DE2 (right panel) donor. Low magnification images of pancreas sections (left inset panels, 1 cm scale bars) with each islet annotated as being an uninflamed Insulin-containing islet (ICI) – red; inflamed, defined as >15 CD45+ immune cells/ islet, ICI – green; inflamed insulin-deficient islet (IDI) – blue; or uninflamed IDI – pink. T1DE1 donors have evidence of more inflammation (green and blue islet annotations) and fewer residual insulin-containing islets (red/green islet annotations) than individuals with T1DE2. The higher magnification images (right inset panels, 500 µm and 100 µm scale bars) demonstrate the presence of peri-insulitis and invasive insulitis in ICIs of a T1DE1 donor, and an example of low level peri-insulitis observed in a T1DE2 donor. Further immune and beta cell characteristics of the endotypes are described in the boxes.

Collating data from EADB and nPOD to examine the presence of residual beta cells reveals proportionally fewer insulin-containing islets (ICIs) in donors diagnosed at age <7 years at the onset, with residual beta cells declining rapidly beyond this. In contrast, those diagnosed older (age ≥13 years) tend to have more residual ICIs at onset which can be preserved for many years post-diagnosis (Leete *et al.* 2016, Carr *et al.* 2022). Encouragingly, these data mirror circulating C-peptide levels observed in living individuals with comparable age-at-onset and disease duration (Carr *et al.* 2022), suggesting that C-peptide will be a valuable window into the pancreas in future intervention trials. The presence of insulitis (accumulation of infiltrating immune cells at the islet) is relatively rare in T1D pancreata (particularly with disease duration >1 year), being described as an ‘elusive lesion’ by Peter In’t Veld (In't Veld 2011). In those with a disease duration of <1 year, insulitis is more commonly found in those diagnosed young, highlighting again the importance of studying young-onset, short-duration donors to improve our understanding. Studies of short-duration donors reveal two forms of the disease, or endotypes: T1D Endotype 1(T1DE1) and 2(T1DE2) (Leete *et al.* 2016) (Fig. 3). These endotypes differ according to insulitis severity, infiltrating immune-cell profiles (particularly B-lymphocytes), the proportion of the residual beta cells and proinsulin localisation within the beta cells. T1DE1 is associated with young age-at-onset (age <7 years) and a rapid loss of circulating insulin, whereas individuals diagnosed ≥13 years of age (T1DE2) often retain detectable C-peptide long after diagnosis and retain normal proinsulin localisation in most islets. Individuals diagnosed later tend to maintain better blood glucose control and develop fewer complications, which aligns with these pancreatic studies where more beta cells are preserved. Interestingly, in these T1DE2 donors, there is evidence that beta cells may be defending themselves against the immune attack, through the upregulation of non-classical class I HLA molecules, such as HLA-E, -F and -G and the checkpoint inhibitor PD-L1 (Richardson *et al.* 2016, Wyatt *et al.* 2019, Colli *et al.* 2020*a*). These can all negatively regulate, or inhibit, immune cells and may protect beta cells from bystander effects of infiltrating cells and reduce auto-reactive T-cell killing. This could be informative for beta cell replacement strategies in the future.

Carmella Evans-Molina opened her lecture on ‘in vivo beta cell function in pre-clinical T1D diagnostics’ by discussing the analysis of individuals with autoantibody positivity from cohorts such as the TrialNet Pathway to Prevention study and Diabetes Prevention Trial of T1D (DPT-1) (Ferrannini *et al.* 2010, Evans-Molina *et al.* 2018), which suggests there are several predictable metabolic checkpoints that are observed during the progression of T1D. Initially, there is a loss of early C-peptide responses, as well as reduced C-peptide area under the curve responses during oral glucose tolerance tests (OGTT) that can be detected up to 6 years prior to the onset of Stage 3 T1D (Evans-Molina *et al.* 2018). An important caveat is that individuals from these cohorts are identified as antibody positive in cross-sectional screening, so their exact timing of seroconversion is unknown. Nonetheless, during a second phase that continues until approximately 2 years before diagnosis, there is relative stability in OGTT C-peptide measures (Evans-Molina *et al.* 2018). Beginning about 2 years before diabetes onset, there is evidence of rising glycaemia and declining C-peptide secretion. Metabolic deterioration accelerates 1 year to 6 months prior to Stage 3 T1D onset, with marked declines in beta cell glucose sensitivity, rate sensitivity and potentiation coupled with decreased insulin sensitivity, and markedly rising blood glucose levels (Ferrannini *et al.* 2010, Evans-Molina *et al.* 2018). Furthermore, data published following the symposium revealed elevations in blood glucose prior to the appearance of islet autoantibodies (prior to stages 1–2) in children with a high genetic risk score for T1D (Warncke *et al.* 2022).

Dr Evans-Molina went on to consider age as an important modifier of T1D risk, as it is well-accepted that age is inversely associated with the risk of developing T1D in those with autoantibodies (Anand *et al.* 2021). Whether age influences patterns of C-peptide decline before and after the onset of Stage 3 disease is a critical question, especially as it relates to the selection of individuals for disease-modifying interventions and the timing of these interventions during periods of active, accelerated disease. In a recent analysis of DPT-1 data, Evans-Molina showed that younger and older progressors to T1D had a similar rate of decline of both beta cell glucose sensitivity and insulin sensitivity prior to the onset of Stage 3 T1D. This stands in contrast to the patterns observed after the onset of Stage 3 T1D where children manifest a more rapid loss of C-peptide following clinical diagnosis (Greenbaum *et al.* 2012, Hao *et al.* 2016). Consistent with these findings, measurable serum C-peptide and histologic evidence of residual insulin-containing islets in long-duration disease are less likely to be observed in individuals with younger age of onset (Davis *et al.* 2015, Lam *et al.* 2017, McKeigue *et al.* 2019, Oram *et al.* 2019).

### The role of T1D candidate genes on beta cells – from genetics to new therapies

John Todd, in his lecture entitled *‘*Genes to clinic in type 1 diabetes’*,* showed that the candidate gene GLIS3 is clearly a beta cell fragility gene acting particularly in early life, predisposing to T1D in individuals diagnosed under the age of 7. Of note, the gut microbiome encodes functional insulin cross-reactive mimotopes of insulin that may promote tolerance to insulin and tolerance to similar bacterial proteins, for example, transketolase. Particularly in DR3/4-DQ8trans dimer-positive individuals, anti-insulin T cells escape from the thymus in the first months of life, especially during weaning (6 months onwards, 3 months before insulin autoantibodies appear). Translation of genetic information to the clinics should consider the following points: (i) Interval dosing of low-dose interleukin-2 (IL)-2 in children is safe and provides a steady-state increase in Tregs. If the Oxford IL-2 trial (ongoing) shows positive results, efforts to bring IL-2 into T1D prevention trials, and production of a non-invasive form of IL-2 should be commenced, including using IL-2 as maintenance therapy; (ii) healthy weaning should be promoted (especially in families with T1D and families in which anti-islet autoantibody screening has identified an autoantibody-positive child); (iii) healthy living during pregnancy could reduce the incidence of T1D; (iv) reduction of childhood obesity could reduce T1D incidence by ~30%; (v) promotion of gut microbiota and gut epithelial health could prevent T1D.

Lorenzo Pasquali addressed the theme ‘Inflammation and regulatory landscape of beta cells provides new insights into the genetics of type 1 diabetes’. He explained that the exposure of human islets to a pro-inflammatory environment causes profound changes in the beta cell gene expression and regulatory landscape. This process results in the activation of distal cis-regulatory elements bound by immune-responsive and tissue-specific TFs. Activation of these noncoding regulatory elements is coupled with 3D chromatin conformation changes enabling contact with their target gene promoters. Beta cell cytokine responsive enhancers harbour T1D-risk variants, suggesting a new mechanism whereby the variants act at a beta cell level but are functional only upon islet cell perturbation, for instance, as mediated by pro-inflammatory cytokines (Ramos-Rodríguez *et al.* 2019, Eizirik *et al.* 2020). Exploring these relationships could open an avenue to the identification of new T1D candidate genes acting at the beta cell level.

Single-cell approaches allow the detection of a heterogeneous beta cell response to the proinflammatory environment which could in turn inform on a specific, targetable, beta cell sub-population. Moreover, single-cell analyses may uncover a cell-type-specific noncoding regulatory response to cytokines; indeed, cellular responses may be islet beta cell-type specific, which might capture cues of the molecular basis of intrinsic beta cell fragility, as compared to other islet cell populations.

Closing the session, Decio L Eizirik discussed ‘From candidate genes to new therapies for T1D – the TYK2 story’, a project developed in close collaboration with Carmella Evans-Molina. He described that a challenge for studies on risk polymorphisms that may contribute to T1D is how to translate this knowledge into new therapies for the disease. Eizirik has previously described that 80% of the candidate genes for T1D are expressed in human islets (Eizirik *et al.* 2012), a finding also observed in the target tissues of other autoimmune diseases (Szymczak *et al.* 2021). He next compared the gene signatures induced by the cytokine interferon alpha (IFNα) (an ‘early cytokine’ that may contribute to three key features of the histology of human islets in T1D, namely upregulation of HLA class I, induction of ER stress and apoptosis) against the signatures observed in FACS-purified human beta cells obtained from patients affected by T1D and then ‘mined’ the common genes detected against gene signatures induced by a panoply of drugs in other cell systems (Colli *et al.* 2020*b*). This indicated that inhibition of TYK2, a kinase that transduces the early signals of IFNα and is a candidate gene for T1D (polymorphisms that decrease TYK2 signalling decrease disease risk), could revert the IFNα-induced signature. Additional testing of TYK2 inhibitors, done in collaboration with the Evans-Molina group, including agents already in an advanced state of evaluation for the treatment of other autoimmune diseases, indicates that TYK2 inhibition protects human beta cells *in vitro* against IFNα alone or in combination with IL-1β (Coomans de Brachène *et al.* 2020) and has clear beneficial effects in two models of autoimmune diabetes in mice (unpublished data). Importantly, TYK2 deletion in stem-cell-derived beta cells protects these cells against both IFN and CD8+ T-cell-induced damage (Chandra *et al.* 2022). As a whole, these data suggest that TYK2 inhibition is potentially an interesting approach to protect pancreatic beta cells in the early stages of T1D.

### Beta cell stress in T1D

Heiko Lickert discussed ‘New insights into beta cell failure and recovery’. He described that beta cells are heterogeneous in health and disease and understanding the underlying mechanisms may allow to protect or even regenerate a functional beta cell mass (Bader *et al.* 2016, Roscioni *et al.* 2016, Yoshihara *et al.* 2020). Using an scRNA-seq-based evolutionary comparison of healthy mouse, pig and human islets revealed that alpha- and beta cell heterogeneity is conserved among species (Tritschler *et al.* 2022). Interestingly, isolated human islets across several healthy donors showed mature, immature, ER stressed and MHC classes I and II/autoantigen high-expressing clusters of beta cells, suggesting that the isolation procedure induces stress and unleashes beta cell plasticity. Streptozotocin (STZ)-induced diabetes and sustained hyperglycaemia for 100 days combined with scRNA-seq of mouse islets identified markers and pathways of beta cell dedifferentiation (Sachs *et al.* 2020). Single and combinatorial pharmacology revealed that exogenous insulin directly acts on beta cells, reducing glucotoxicity and contributing to restore beta cell identity and function. Interestingly, glucagon-like peptide 1 receptor (GLP-1R) can be targeted to deliver a GLP-1-oestrogen conjugate to beta cells to induce a nuclear response and protect beta cells from ER stress-mediated cell death, avoiding the side effects of oestrogen on female tissues (Sachs *et al.* 2020). It is well known that insulin/IGF signalling is essential for beta cell survival, proliferation and function and that beta cell insulin/IGF resistance causes overt diabetes (Goldfine & Kulkarni 2012, Jain *et al.* 2022). The discovery of the insulin inhibitory receptor in beta cells may allow specific sensitisation of insulin/IGF signalling in islet beta cells to trigger proliferation and survival (Ansarullah *et al.* 2021). In conclusion, understanding paths and mechanisms of beta cell stress, loss of identity and dedifferentiation and identifying and validating druggable molecular targets may allow to protect and/or regenerate beta cells in type 1 (and 2) diabetes.

Raghu Mirmira addressed the search for ‘Circulating biomarkers of beta cell stress and death’. In early T1D, beta cells are subjected to stresses such as inflammation, viral infections, hypoxia/ischemia and impaired nutrient handling as a result of insulin deficiency (Eizirik *et al.* 2009, Wilcox *et al.* 2016, Chen *et al.* 2017). Under these conditions, beta cells trigger emergency responses, including the ‘integrated stress response (ISR)’, which is highlighted by the activation of one or more of four kinases (PERK, HRI, PKR, GCN2) that lead to the phosphorylation of the translation factor eIF2α (Taniuchi *et al.* 2016). Phosphorylated eIF2α blocks the translational initiation of many mRNA transcripts in an attempt to divert energy expenditure toward cellular recovery processes (Muaddi *et al.* 2010). If the block is not alleviated, then the cell is bound for an apoptotic fate. Thus, like many emergency responses in the cell, the ISR balances the cellular fate. During the ISR, translationally inhibited mRNAs are compartmentalised into discrete processing bodies (P-bodies) and non-membranous inclusions known as stress granules (SGs) (Kedersha *et al.* 2013, Wheeler *et al.* 2016, Namkoong *et al.* 2018). PBs function in normal RNA decay, though this idea remains somewhat controversial (Luo *et al.* 2018). SGs, by contrast, are induced by cellular stressors (e.g. inflammation, viral infections) (Decker & Parker 2012) and function as cellular signalling ‘nodes’, whereby their predominant fate (recycling of their contents to translating ribosomes, diversion to lysosomes for degradation or fusion into endosomal cycling cascades) may ultimately determine the cell survival. Pancreatic tissue sections from autoantibody-positive non-diabetic individuals showed an increase in general SG formation in beta cells, whereas in longstanding T1D, SGs were significantly reduced. A perspective has been emerging that SGs may intercept other intracellular pathways (Buchan & Parker 2009, Buchan *et al.* 2013, Kedersha *et al.* 2013), including endosomes, which are eventually released as extracellular vesicles (EVs) (Baixauli *et al.* 2014, Jimenez *et al.* 2019). All EVs carry cell/stress-specific cargo that includes proteins, nucleic acids and lipids. The inclusion of SGs into forming endosomes and EVs (Jimenez *et al.* 2019) may augment a mechanism by which a cell’s ‘state of emergency’ can be communicated extracellularly. Dr Mirmira’s data demonstrate that the triggering of the ISR results in the inclusion of insulin mRNA within SGs. In humans with new onset T1D, insulin mRNA is detectable in circulation, whereas in longstanding T1D (where beta cells are few or dysfunctional), insulin mRNA is undetectable. These findings hold the promise that interrogation of EVs from individuals at risk for T1D might uncover mRNA species that reflect ongoing stress within beta cells. Such knowledge has the potential to identify high-risk individuals for whom intervention might prevent subsequent T1D.

Anath Shalev discussed the approaches her group is developing regarding ‘Reducing beta cell stress – clinical potential’. Recent clinical trial data (Ovalle *et al.* 2018, Xu *et al.* 2022) demonstrate that continuous oral use of the approved blood pressure medication, verapamil, in subjects with T1D delays loss of beta cell function and lowers insulin requirements for at least 2 years post-diagnosis. Verapamil downregulates the detrimental protein thioredoxin-interacting protein (TXNIP) and promotes an anti-oxidative, anti-apoptotic and immunomodulatory gene expression profile in human islets. Oral verapamil treatment reverses T1D-associated increases in serum chromogranin A (a T1D autoantigen) and in proinflammatory factors (IL-21 levels and T-follicular helper cells). This is the first indication that verapamil may also affect the autoimmunity of T1D in addition to its effects on beta cell survival. As such this could also help explain why verapamil is successful even without additional bona fide immunomodulation. Importantly, since verapamil is very well tolerated and its effects are not dependent on the presence of insulitis, testing it for the prevention of T1D in at-risk subjects seems warranted and would provide an attractive risk–benefit ratio. In addition, global longitudinal serum proteomics analysis revealed that chromogranin A, which can also be detected by a simple clinical blood lab test, provides a promising novel marker to help monitor successful therapy or disease progression in T1D. These studies provide an example of how findings in human islets and diabetes mouse models other than the non-obese diabetic (NOD) model of T1D can be successfully translated into clinical trials.

In the last lecture of the session, Alberto Pugliese discussed *‘*Exploring circulating miRNAs as biomarkers of disease progression, beta cell loss and stress’*.* miRNAs are critical regulators of gene expression and modulate pathways relevant to T1D. During the past decade, studies have explored associations of circulating miRNAs with T1D and to improve prediction of T1D risk and progression, including the decline of insulin secretion. Studies differed in design, cohorts, assays employed to measure miRNA levels, number of miRNAs measured, sample types tested (serum, plasma, rarely vesicles) and data analysis approaches. Despite the above mentioned differences, a review of published literature shows that among miRNAs whose circulating levels were associated with T1D/islet autoimmunity or disease progression, 35 were reported by more than 2 independent studies (range 2–8 studies reporting associations), including 12 miRNAs reported by at least 3 independent studies. These observations suggest that circulating miRNAs can be exploited as disease biomarkers with further advances and validation. This will require access to replication cohorts, and ideally, future studies should examine longitudinal samples. Ongoing studies are now relying on RNAseq-based platforms to examine the levels of higher numbers of miRNAs (extending to the 2000 range), and the sample volume requirement is down to microlitres, with minimal impact on existing repositories. It is possible that several of the earlier associations, based on RT-PCR assays that assessed a more limited number of miRNAs, may not be replicated due to differences in assay format and yet there is potential for the discovery of more and stronger associations. Reconciling RT-PCR and RNAseq data may be challenging but should be attempted and standardised assays should be developed for the clinical setting. Studies of miRNA responses in the context of disease-relevant metabolic/immunological stimulations would be helpful to better understand the biological impact of miRNAs, to unveil dysregulation of miRNAs that may not be evident in the steady state and to improve our understanding of the cellular origin of miRNAs in the circulation. To this end, initial studies showed the feasibility of these approaches, for example, by measuring changes in miRNA levels in patients, *in vivo*, during a mixed-meal tolerance test and in parallel during glucose-stimulated insulin secretion from isolated pancreatic tissue.

### Beta cell replacement and protecting beta cells from the immune system

**Figure 4:**
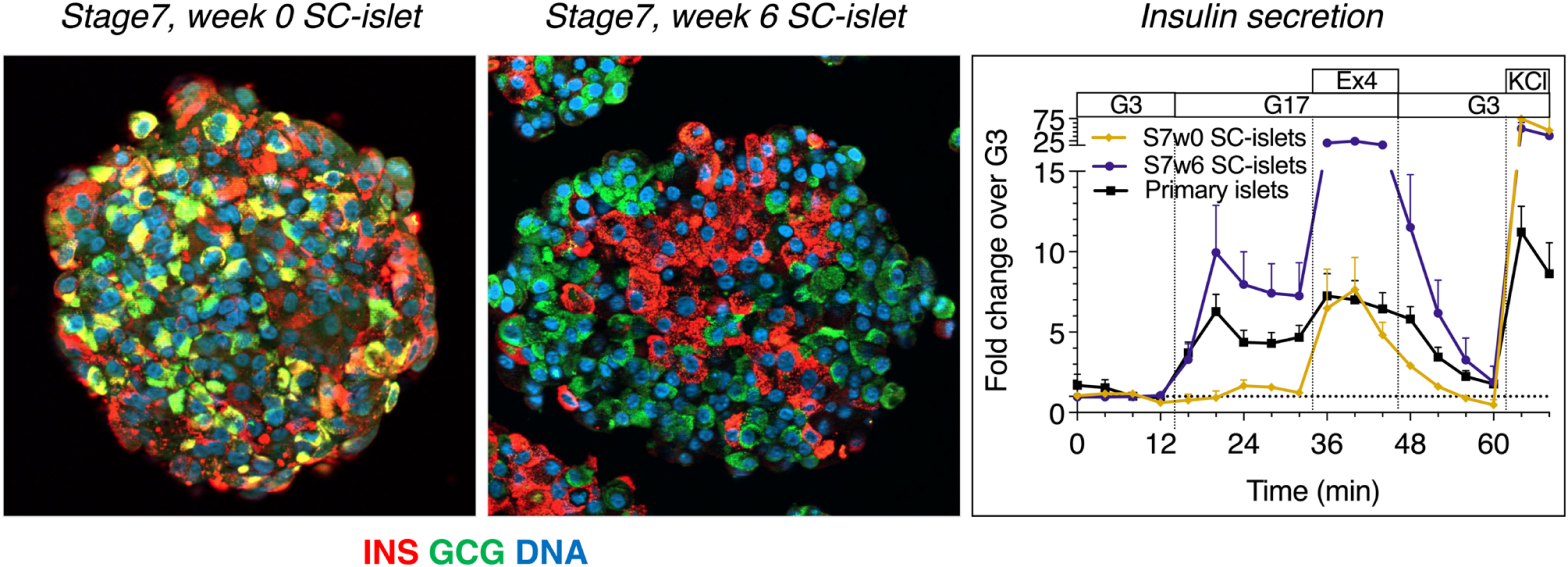
Human pluripotent stem cell-derived islets (SC islets). Double immunostaining for insulin and glucagon in the immature SC islets (stage 7, week 0) shows many double-positive cells. In the mature stage, the hormone-positive cells are clearly defined as separate populations in an architecture that resembles the human islet. Dynamic insulin secretion was studied with perifusion. The immature SC islets do not respond to high glucose, although they are able to respond to exendin-4 and membrane depolarisation induced by potassium chloride (KCl). In contrast, the mature-stage SC islets show a high response to glucose. (Data from Balboa *et al.* Nature Biotech 2022. Figure prepared by Väinö Lithovius.)

Timo Otonkoski addressed the question ‘Beta cells from ES- and iPS-cells: how close are we to endogenous beta cells?’ by presenting a comprehensive characterisation of human pluripotent stem cell-derived islets (SC islets) and primary human islets, focusing on their structure, function (both *in vitro* and *in vivo* following murine implantation), gene expression and metabolism as described in recent reports (Balboa *et al.* 2022, Barsby *et al.* 2022). Using a modified seven-stage differentiation protocol, he showed that when SC islets are given sufficient time to mature *in vitro*, they develop into islet-like aggregates that consist of nearly 100% endocrine cells (40–50% of both alpha and beta cells, 5% delta cells and 5–10% enterochromaffin-like cells, which may in fact represent endocrine progenitors) (Fig. 4). The SC islets present dynamic glucose-stimulated insulin secretion that closely matches adult islet controls, except for a somewhat lower extended second phase. When transplanted into immunodeficient mice, the SC islets become highly vascularised and innervated. However, as compared with implanted adult islets, there is a longer lag time before SC islets fully control the glycaemia of diabetic mice. The SC islets demonstrate signs of metabolic immaturity, for example, strong responsiveness to exogenous pyruvate and lower coupling of mitochondrial respiration to glucose-induced insulin secretion. However, these metabolic differences appear to quickly decrease *in vivo*, after implantation in mice, in agreement with transcriptomic data of maturation-associated genes and pathways. Overall, SC islets represent a valuable source of human pancreatic endocrine cells that can be effectively used both for the modelling of pathogenic mechanisms behind various types of diabetes (Balboa *et al.* 2021) and for the development of cell replacement therapies for insulin deficiency.

In the lecture entitled ‘Stem cell to beta cell: state of the art and therapeutic potential’ Louise Winkel provided an industrial perspective on stem cell to beta cell technology, including a discussion of regulatory, manufacturing and logistical challenges that remain to bring a stem-cell-based therapy to the clinic. Novo Nordisk has been active in stem cell research for over 20 years. In late 2018, Cell Therapy R&D was established as an independent unit and has since grown to cover the full pharmaceutical value chain, from research to commercial, including a good manufacturing practice (GMP) manufacturing site in Fremont. In addition to several exploratory projects, three projects are moving towards the clinic: Parkinson’s disease, chronic heart failure and T1D, all but the latter in close collaboration with external partners.

The protocol for generating functional islet-like clusters from pluripotent stem cells has been developed internally over the past 14 years and is now matured to generate thoroughly characterised, functional cells that reproducibly reverse diabetes in pre-clinical models. This has been a major achievement not only accomplished by Novo Nordisk but also by several other groups in this field. However, it is ‘only’ the beginning of even bigger challenges to solve from an industrial perspective. Setting aside the major and complex challenges of the route of administration and protection from the immune system in people with T1D, upscaling and development of processes and assays that support large-scale GMP production, quality control and distribution beyond investigational new drug (IND)-enabling studies and Phase 1/2 trials is pivotal to the success of any cell-based therapy.

The development, delivery and commercialisation of stem cell-based therapies differ significantly from traditional drug development. Unlike protein- and peptide-based therapies, few standardised procedures are currently established across the industry or at the regulatory level. Each company must develop and mature both capabilities and processes as projects evolve, which requires very close collaboration across the research and development value chain. In addition, each cell type has different requirements and reliable delivery of cells into the body is extremely complex.

In summary, the field needs to focus on quality, scalability and cell delivery to accomplish our shared goal of helping people living with serious chronic diseases with safe and efficacious cell-based therapies.

Shareen Forbes presented work on ‘islet transplantation and mesenchymal stem cells’. Islet transplantation is of proven efficacy in T1D, reducing hypoglycaemia, stabilising blood glucose levels and improving the quality of life. However, there is a limited supply of donor pancreata and each recipient requires islets from two to three donors to substantially impact on the glycaemic control. Rates of insulin independence are only 17% in the UK, there is attrition in graft function over time and the scarcity of donor pancreases for transplantation further compounds the problem. Islets are avascular when transplanted into the liver, rendering them vulnerable to hypoxia and immune-mediated apoptosis and reliant on angiogenesis and vasculogenesis to engraft. As such, >60% of the transplanted islets die in the first 48 h post-transplant. Treatments to ameliorate this loss are urgently required.

Mesenchymal stromal cells (MSCs) are found in a number of organs and support cells within tissues. They are defined by the presence and absence of specific cell surface markers, are adherent to plastic and are able to differentiate into fat, bone and cartilage. MSCs from different sources are heterogeneous but can home to sites of inflammation and secrete immunomodulatory and anti-inflammatory cytokines as well as growth factors that are pro-angiogenic. MSCs are currently in Phase 3 clinical trials and have shown efficacy in a number of settings including acute graft vs host disease when given at a high dose intravenously (>1 × 10^6^ MSCs). They are therefore potential candidates to improve islet engraftment and long-term graft function. Dr Forbes described their recent efforts to manufacture umbilical cord-derived human MSCs to GMP grade, with gene expression studies demonstrating anti-inflammatory, immunomodulatory and pro-regenerative properties (Forbes *et al.* 2020)). In a diabetic immunodeficient mouse model followed for 16 weeks, the Forbes team has shown superior glycaemic control, greater stimulated C-peptide concentrations, increased graft vascularity and delayed rejection with umbilical cord-derived MSCs plus islets vs islets alone (Forbes *et al.* 2020).

A current barrier to the translation of this therapy into humans is the demonstration of a homogeneous cryopreserved-banked MSC product, which is efficacious and safe at this proposed dose in islet transplantation. This would enable this allogeneic product to be thawed on demand and infused with islets into humans. Such a strategy would allow islet grafts to function better for longer periods and allow pancreases to be distributed to more people.

Kevan Herold provided an up-to-date review of ‘Immunotherapy and interventions in pre-diabetes and diabetes’. A number of immune interventions have been successfully trialled for the treatment of T1D in those with recent onset disease (Herold *et al.* 2002, 2005, 2013). Most recently, T1D clinical onset has been delayed in some individuals at risk for the diagnosis but who are free of symptoms for extended periods of time (Herold *et al.* 2019). However, even with the successes, the end of the disease has not yet been achieved, and further work is needed. The following observations and conclusions, based on the experience from clinical and preclinical studies, may help guide this work: first, there appears to be a window for successful interventions with the agents that have been able to modulate the natural disease course. This appears to be in the peri-diagnosis period (pre- and immediately post-diagnosis), which is thought to be characterised by active immune killing of beta cells. The reasons why this period is opportune have not been defined but it may relate to the susceptibility of immune cells to modulation. Second, the effects of immune therapies have not been permanent – in most cases, tolerance has not been achieved. With B cell depletion with anti-CD20 mAb (Pescovitz *et al.* 2009), which can modulate the decline in beta cell function when it is given to patients with new-onset disease, the recovery of B cells in the peripheral blood includes the recovery of autoreactive B cells (Chamberlain *et al.* 2016). Likewise with anti-CD3 mAb, autoreactive T cells are not permanently depleted. Recent preclinical and translational studies have highlighted the stem-cell-like features of autoreactive CD8+ T cells in T1D suggesting that these cells are resistant to elimination and may persist even after immune therapy. It suggests that rather than depleting the autoreactive repertoire, achieving tolerance will require an environment or ligands that prevent these autoreactive cells from reactivation. Finally, recent studies have described the contribution of beta cells themselves to their own demise (Rui *et al.* 2021, Mallone *et al.* 2022, Piñeros *et al.* 2022). They are an important source of inflammatory mediators that may recruit and activate autoreactive cells. Blocking these mechanisms in beta cells may solve both the problem of immune cell activation and beta cell killing.

With the progress of immune therapies, a new frontier is opened in which the goals for the treatment are directed towards the maintenance of the efficacy seen with single agents, timing of therapies, combinations of agents that may target immune and beta cells and the selection of patients who are most likely to respond to interventions. Much like the first descriptions of success with cancer immune therapy, progress in the field is likely to expand rapidly.

*Coda: Since the symposium the US Food and Drug Administration have approved the use of* *Teplizumab (anti-CD3) injection to delay the onset of stage 3 T1D in patients >8 years of age with stage 2 T1D.*

### Measuring success in replacement/regeneration in pre-clinical and clinical models

Peter Senior opened this session by discussing ‘Clinical Assessment of Islet Cell Function’ from the standpoint of human islet transplantation. The University of Alberta has performed 729 islet transplants on 312 individuals since 1999 with a goal of improving both ‘metabolic health’ and ‘mental health’. Factors such as reducing glucose variability as well as reducing or abolishing hypoglycaemia and the fear of hypoglycaemia are important in improving ‘mental health’ beyond average glucose or HbA1c levels. Frequent assessments of islet cell function are required following islet transplantation to identify the need for additional islet loading (second or third islet transplants) and/or evidence of rejection. More invasive/extensive assessments of islet function provide more information, but a balance has to be struck with the patient burden of repeated assessments. In addition, stimulated C-peptide levels are very dependent on the stimulus such that glucose-potentiated arginine stimulation (with glucose = 360 mg/dL) could release three to four times as much C-peptide in islet transplants recipients than arginine alone (Rickels *et al.* 2005). The formulae to assess insulin sensitivity (e.g. HOMA or homeostatic model assessment) and to relate C-peptide to glucose (e.g. Suito Index) improve clinical correlation but are generally developed in the non-insulin-treated healthy population and may have limitations in the clinic population. More complex composite scores such as the BETA2 score which includes insulin dose and HbA1c as well as glucose and fasting C-peptide (Ryan *et al.* 2005, Forbes *et al.* 2016) are more convenient to use in clinical practice and correlate well with clinical outcomes (insulin independence) and invasive measures (*r*^2^ = 0.69) (Senior *et al.* 2020*b*, Lam *et al.* 2022). Continuous glucose monitoring (CGM) is required to capture variability and can show improvement following islet transplantation compared to hybrid closed-loop insulin therapy (Senior *et al.* 2020*a*). Further studies are required to indicate if this correlates well with ‘mental health’ in addition to ‘metabolic health’.

Michael Brehm discussed advancements in ‘Humanized models to understand the crosstalk between beta cells and the immune system’. Generating such models is a challenging task with several components. First, it is necessary to delete elements of the mouse immune system to ensure that any response to islet grafts is ‘human’ in origin. Deletion of the common gamma chain in NOD scid gamma (NSG) and NOD-Rag1^null^ (NRG) mice impairs signalling through multiple key cytokines (IL-2, IL-4, IL-7, IL-9, IL-15 and IL-21) resulting in the lack of T, B and natural killer (NK) cells (Ishikawa *et al.* 2005, Shultz *et al.* 2005, Pearson *et al.* 2008). Secondly, the deletion of murine insulin secretion allows the detection of the function of any transplanted islets via monitoring glucose levels. Such ‘hyperglycaemic’ mice can be generated either using the beta cell toxin streptozotocin or introducing the Akita insulin 2 gene mutation, which has a dominant negative effect on insulin production by impairing proinsulin folding in all forms of insulin (Pagliuca *et al.* 2014). Inducible beta cell depletion can be introduced using the rat insulin promotor-diphtheria toxin responsive element (RIP-DTR). Thirdly, the human immune system needs to be engrafted. This has been done by transferring either human peripheral blood mononuclear cells (PBMC) or haematopoietic stem cells (HSC). Unfortunately, such mice do not have a long lifespan due to Xeno-graft vs host disease, but this can be reduced by the deletion of mouse MHC class I and II without irradiation. NSG RIP-DTR MHC-DKO mice reconstituted with 50 million human PBMCs given intraperitoneally that receive diphtheria toxin reject a human islet allograft as shown by rising glucose levels (Brehm *et al.* 2019). Islet organoids derived from human pluripotent stem cells can be used in place of islet tissue and these systems have been used to demonstrate that overexpression of programmed death-ligand 1 (PD-L1) protected the organoids from rejection (Yoshihara *et al.* 2020). This platform has also been used recently by Doug Melton’s group to show that interferon-gamma signalling plays a major role in the rejection of stem cell-derived islets and this can be ameliorated to some degree by the deletion of CXCL10 in the stem cells (Sintov *et al.* 2022). Currently, work is underway to enhance the development of the engrafted human innate immune cells by expression of cytokines such as human IL-15 or human fms related receptor tyrosine kinase 3 (FLT3)-ligand. In addition, expression of mouse thymic stromal lymphopoietin has been shown to restore lymph node development in these composite mice, which is otherwise lacking.

James Cantley discussed ‘Preclinical models and assessment of islet function’, beginning by reviewing rodent models for T1D, including STZ-induced insulin-deficient diabetes, dosed to induce acute (Cantley *et al.* 2013) or progressive (Cantley *et al.* 2011) beta cell decline, along with the NOD/ShiLtJ mouse model of T1D. NOD mice are widely used in preclinical T1D studies with examples of successful translation to human trials including anti-CD3 immunotherapies (Herold *et al.* 1992, Chatenoud *et al.* 1994, Herold *et al.* 2019) and stem cell-derived pancreatic cell grafts (Agulnick *et al.* 2015, Ramzy *et al.* 2021, Shapiro *et al.* 2021), and some failures to translate. Despite steady improvements in pre-clinical study design, adopting many of the principles of clinical trials (Atkinson 2011, Grant *et al.* 2013, Gill *et al.* 2016), current pre-clinical endpoints are typically static-fed blood glucose measurements with a diabetes diagnostic threshold of >250 mg/dL. CGM has revolutionised the clinical management of diabetes, whilst providing new insights into glycaemic variability and its relationship with beta cell function (Gibb *et al.* 2020, Rickels *et al.* 2020, Carr *et al.* 2021, Yeşiltepe-Mutlu *et al.* 2021), risk of disease progression (Steck *et al.* 2022) and diabetes complications (Wilmot *et al.* 2021). Recent advances in telemetry have enabled remote CGM in rodents via surgically implanted carotid glucose sensors, providing new insights into glycaemic variability (Lo Martire *et al.* 2018), revealing marked daily glucose fluctuations during the prediabetic phase in NOD mice (Korstanje *et al.* 2017) and following minimal-mass islet transplantation in STZ-induced diabetic rats (King *et al.* 2017). This has important implications for binary pre-clinical study endpoints utilising the 250 mg/dL threshold, with a risk of false negatives and positives in prevention or reversal studies. Strategies for quantifying glycaemic variability during the pre-diabetic and diabetic phases were considered, such as rodent-adapted time-in-range, % coefficient of variation (%CV) or interquartile range, providing a graded assessment of disease progression and pre-clinical efficacy, whilst mirroring human glycaemic metrics. Barriers to wider implementation of preclinical CGM include expensive and invasive telemetry implants, with further innovation in rodent CGM, application of human patch systems to larger model species or better use of static blood glucose measurements needed to circumvent this.

In the second part of the lecture, Dr Cantley discussed the challenges of assessing beta cell mass and 3D structure of the rodent pancreas using conventional histology. A new Insulin1-tdTomato knock-in reporter mouse generated by the lab was presented which, combined with tissue clearing approaches and Mesolens imaging (Battistella *et al.* 2022), has enabled high-resolution imaging of total beta cell mass in the intact pancreas, whilst providing a proportional readout of insulin1 promoter activity.

### What is missing for translation?

Steven Parks is a member of the Diabetes UK administrative team and was diagnosed with T1D aged 3 years. He shared key reflections on the translational gaps that need to be filled to address the needs of patients and their families. In particular, he emphasised that the needs differ at different ages – for example, between young children, teenagers and older adults. The sudden diagnosis of T1D after a very short (weeks) prodrome was a huge shock to his family and better markers able to monitor the decline in beta cell function in pre-diabetes, along with immune and genetic markers to predict who will develop T1D, are important to better prepare and therefore reduce the impact of diagnosis for families. At all stages, the idea of being immunosuppressed long-term as part of therapy raises concerns and risks need to be minimised, for example, by targeting treatments to islet cells. Following diagnosis, Steve confirmed that variability and unpredictability in glucose control is a major stress for people living with T1D. Reducing the complexity and burden of dealing with the different effects of food, exercise and psychological stress on glucose levels is essential. Although the technology of insulin replacement has advanced considerably, many people do not find it a sufficiently discrete, reliable or low burden to engage with it in its current form, especially among teenagers and young adults, emphasising the continued need for interventions such as prevention and stem cell replacement. At all stages of research, from planning to late stages, Steven emphasised the importance of including people living with diabetes and their families to ensure that what appears a good idea to research teams does really meet their needs and comprises an acceptable balance of benefits and risks.

In his lecture entitled ‘Clinical endpoints in beta cell preservation – Competing or complementary therapies?’, Colin Dayan built on Peter Senior’s comments, emphasising the need for improved C-peptide and clinical endpoint assessments in studies of islet cell function. Measurement of the area under the curve (AUC) of insulin/C-peptide over 2 or 4 h following a mixed-meal tolerance test has been the standard measure of beta cell function for 20 years. However, it is well known that beta cells secrete insulin in two phases – a rapid first phase and a slower second phase. The first phase is critical to regulate post-prandial peaks of glucose and reduce variability but occurs within 10–15 min, so it is not well captured by integration of the C-peptide and glucose response over 2–4 h. Consistent with this, there is only a modest correlation between AUC C-peptide or the detected peak C-peptide level (typically at around 90 min) and measures of glycaemic control, for example, HbA1c or CGM parameters (*r* values typically around 0.4) (Buckingham *et al.* 2015). Analysis of pooled data from 18 intervention trials of immunotherapy in new-onset T1D, 6 of which met their primary end point, conducted within the Trial Outcome Markers Initiative (TOMI) hosted by the C-Path Consortium has shown that immunotherapy (vs placebo) has a significant impact on AUC C-peptide at 6 months but not after 3 months. The same is true for insulin doses. By contrast, differences in HbA1c levels are seen at 3 months, suggesting that immunotherapy has an important early impact on beta cell function that is not detected by the AUC metric. Several alternative measures take into account the relationship between glucose and C-peptide levels, as Peter Senior indicated in his presentation, for example, Insulin dose-adjusted A1C (IDAA1c), the Suito Index, the B2 score, and glucose and C-peptide response curves (Sims *et al.* 2021); however, these do not reflect biphasic secretion. The oral minimal model (OMM) developed by Cobelli and colleagues (Cobelli *et al.* 2014, Galderisi *et al.* 2021) can estimate biphasic insulin secretion from the OGTT. The utility of hypoglycaemia as an outcome in clinical trials of beta cell preservation in new-onset T1D is also being explored within TOMI. Dr Dayan went on to describe the use of adaptive platform trials to study multiple combinations of agents (and agents at different doses) for beta cell preservation in a more rapid and efficient manner than with standard randomised controlled trial designs, as illustrated by the T1D Plus platform being developed in the INNODIA network. Work on the most sensitive and rapidly responsive measure of beta preservation will further accelerate these efforts and progress in drug development in this area.

‘The closed loop story – advantages and limitations’was the theme addressed by Charlotte Boughton, including recent advances in this field and the recently published Closed Loop from Onset of T1D (CLOuD) study applying closed-loop therapy from the diagnosis of T1D in children (Boughton *et al.* 2022). Closed-loop insulin pump therapy, in which insulin delivery is linked to CGM via an algorithm, aims to mimic islet function as closely as possible. She emphasised the challenge of glucose variability and unpredictability in insulin requirements that Steven Parks had highlighted, especially in very young children. Data from observation of over 2000 days in 114 different individuals showed that children aged 1–6 have a %CV in daily insulin requirements as high as 46%, as compared to values of around 36% in teenagers and young adults (Dovc *et al.* 2019). Closed-loop therapy reduces rather than increases the burden for patients, with users spending on average 36 min/day on diabetes management. Hyperglycaemia is potentially detrimental to islet cells (‘glucotoxicity’) including impairing beta cell function. It has therefore been hypothesised that improved glycaemic control immediately after diagnosis may improve islet cell function and survival. However, reports to date have produced conflicting results (Shah *et al.* 1989, Buckingham *et al.* 2013). The CLOuD study aimed to address this issue in young people aged 10–17 by comparing the use of hybrid closed-loop therapy with standard management (43% using pump therapy and 68% using CGM by 24 months) starting within 21 days of diagnosis (Ware *et al.* 2022). HbA1c levels were 0.4 percentage points lower at 12 months (6.9% vs 7.3%) and 1 percentage point lower at 24 months (6.9% vs 8.0%) in the hybrid closed-loop group, but no difference was seen in AUC C-peptide levels in the mixed-meal tolerance testing. Participants in the intensive arm used the closed-loop system for 76% of the time, which suggests that it had good levels of acceptability. However, five cases of severe hypoglycaemia and one case of diabetic ketoacidosis still occurred in the closed-loop group emphasising the remaining unmet need for replacement/preservation of islet function.

## Conclusions and roadmap for future research

**Figure 5:**
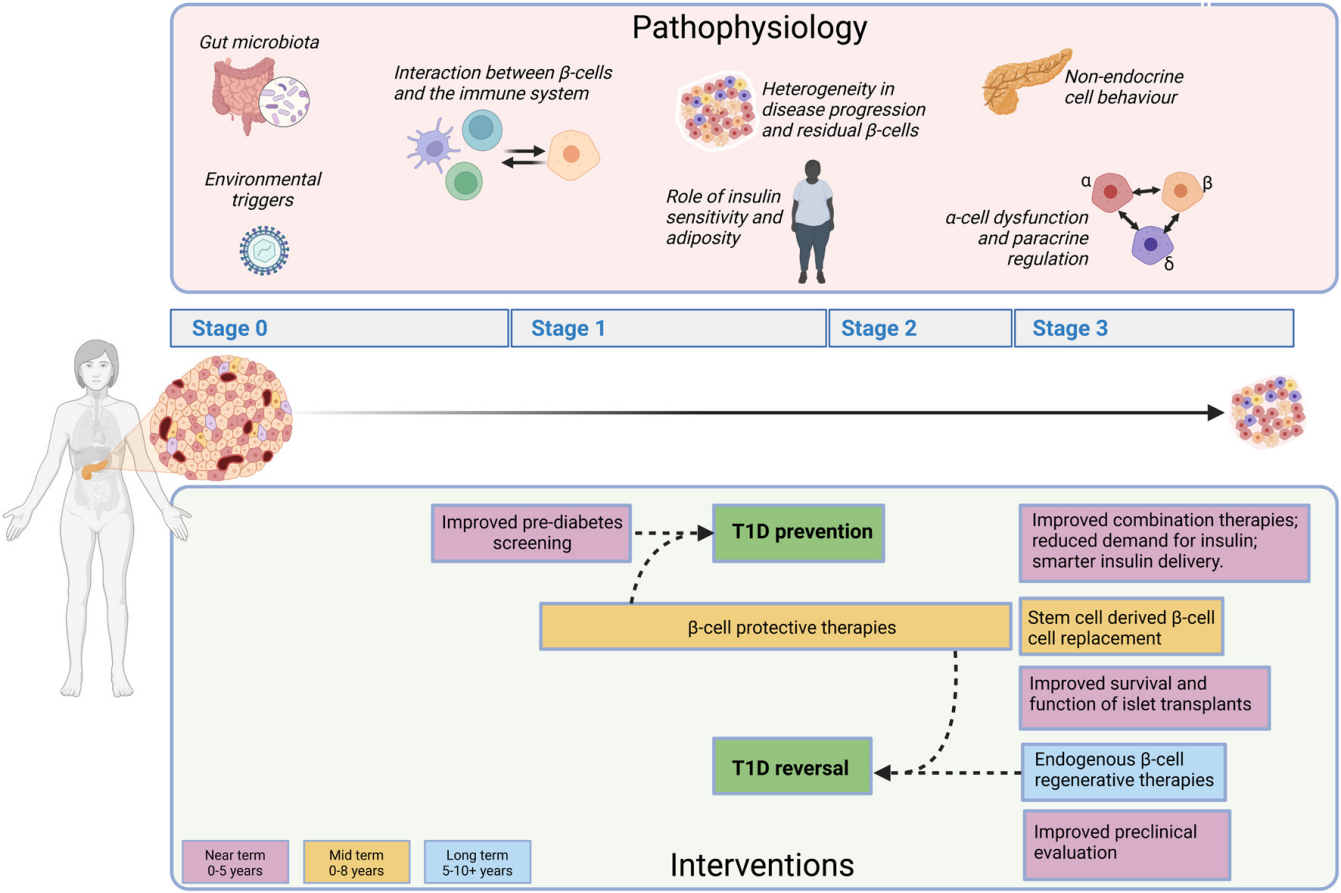
A suggested roadmap for future type 1 diabetes (T1D) research. Summary of priorities for T1D research as described in detail in the main text. Pathophysiology (upper section). Risk factors for T1D involve a combination of genetic and environmental factors, including gut microbiota and viral infection. There is an urgent need for more research into the early stages of disease onset, including environmental triggers and the interaction between beta cells and the immune system. Progress in recent years has revealed marked heterogeneity in the age of diagnosis, disease progression and residual beta cell function in people with T1D. Further research into the factors underlying this heterogeneity is needed to generate deeper insights into disease aetiology, identify prognostic markers and enable personalised treatment strategies. With increased global obesity, including in childhood, more research into the role of obesity, sedentary lifestyle and insulin sensitivity on T1D progression is warranted. A recurring theme in the meeting was the dysregulation of glucagon secretion from pancreatic alpha cells (in spite of the fact that beta cells but not alpha cells are killed in long-term diabetes) and the imbalance of glucagon and insulin in the T1D pancreas. Finally, alterations in the exocrine and ductal system of the pancreas have been observed in T1D. Lower section, interventions. Summary of established and emerging approaches to treat T1D is provided, with estimated timescales to clinical impact (colour coded: pink, near-term 0–5 years; orange, mid-term 0–8 years; blue, long-term 5–10+ years). Improved pre-diabetes screening will support targeted immunotherapy, beta cell preservation and ultimately T1D prevention. Optimised combination therapies, reduced insulin requirements and smarter insulin delivery have the potential to improve clinical care in the near term. Stem-cell-derived cell transplant therapies are in clinical trials with positive early reports and have the potential to improve regulated insulin release in T1D in the mid-term. Likewise, strategies to improve the success of islet transplant therapy have the potential to reduce insulin dependence in the short term. Strategies to regenerate endogenous beta cells have long-term potential to restore regulated insulin secretion in the pancreas which, if coupled to beta cell protective therapies, may reverse T1D. Improvements in preclinical models and protocols can accelerate many of these therapeutic avenues. Figure created with BioRender.com.

A suggested roadmap for future T1D research is summarised in Fig. 5. New imaging technologies applied to pancreatic biobank tissues and islets are transforming our view of the natural history of T1D. They demonstrate that beta and alpha cell changes occur early during pathogenesis, promote new hypotheses on the role of exocrine cells and suggest that a stage 0 T1D may exist (beta cell dysfunction and impaired glycaemic control, in the absence of autoimmunity). Further investigation of the role played by alpha cells in the pathophysiology of T1D, and why beta but not alpha cells are killed in long-term T1D, is clearly warranted.

Histopathological differences between putative T1D endotypes continue to be explored. Invasive insulitis observed in childhood T1DE1 could be associated with alterations in islet basement membrane components and integrity. In contrast, the islet basement membrane appears structurally more intact in older onset T1DE2 with mild insulitis. Alterations in its integrity could account for different rates of disease progression and possibly for the sudden acceleration close to diagnosis after many years of ‘status quo preservation’. In addition, the lack of direct immune-beta cell contact in situations where the islet basement membrane is intact supports soluble factors mediating the dialogue between the two cell types and warrants further investigation. Insulin sensitivity may also play a role in shaping age-related endotypes. Research into the role of gut microbiota in T1D susceptibility, especially during weaning, may provide new insights and potential therapeutic strategies.

The hypothesis of the existence of T1D heterogeneity is starting to move from a phenomenological concept to one supported by novel mechanistic hypotheses. On one side, this invites enhanced efforts in the study of long-term non-progressors to identify disease-protective factors that may point to novel therapeutic targets. On the other, the often advocated immunomodulatory and beta cell-protective combination therapies may also be achieved by single agents targeting both immune and beta cells, for example, JAK or TYK2 inhibitors and verapamil. In addition, studying young organ donors with multiple autoantibodies but without overt hyperglycaemia (stages 1–2) will provide insights into disease pathogenesis and heterogeneity, interactions between the beta cell and immune system and potentially reveal new therapeutic targets.

It is likely that combination therapies with investigational products (reducing insulin dependence) and insulin delivery via closed-loop systems (refining insulin administration) will become standard therapeutic approaches in the near term. The ability of anti-CD3 administration to delay the onset of T1D provides a great source of optimism for the future of immunotherapies to preserve beta cell function. Improved screening and biomarkers to determine T1D risk will support-targeted implementation of these therapies. Although closed-loop systems did not improve the preservation of residual C-peptide in the recent CLOuD trial, it may act as a useful adjuvant for other immunomodulatory and beta cell-protective therapies. New strategies to improve the success of islet transplant therapies, such as encapsulation or co-transplantation with mesenchymal stromal cells, also have the potential to reduce insulin dependence in the near term. Recent progress in the advancement of stem cell to beta cell replacement therapy has clinical potential in the medium term, although regulatory, manufacturing and cost challenges remain. Finally, the development of strategies to drive regeneration of endogenous beta cells remains a long-term research goal which, in combination with recent advances in immunotherapy, could provide stable restoration of a functional pancreatic beta cell mass. Successful and timely development of these therapeutic approaches will require continued improvements in the assessment of glycaemic control/variability and beta cell function metrics as robust clinical/preclinical markers and endpoints. Improved understanding of T1D risk, heterogeneity and identification of prognostic markers will facilitate stratified treatment of the disease.

In summary, this symposium has captured a cross-section of current T1D research, including the aetiology and heterogeneity of T1D along with the current state-of-the-art and clinical potential of treatment strategies, both established, emerging and in development. This research landscape inspires optimism for the future management, treatment and even prevention of T1D.

## Declaration of interest

JC and DE have no competing interests to declare. EL is a former employee of Regeneron Pharmaceuticals and owns company stock. CD has lectured for or been involved as an advisor to the following companies: Novonordisk, Sanofi-genzyme, Janssen, Servier, Lilly, Astrazeneca, Provention Bio, UCB, MSD, Vielo Bio, Avotres, Worg, Novartis. CD holds a patent jointly with Midatech plc.

## Funding

We would like to thank the JDRF and DiabetesUK for sponsoring this symposium. JC is funded by a Steve Morgan Foundation Type 1 Diabetes Grand Challenge Senior Research Fellowship (22/0006505), the Medical Research Council (MR/W019590/1) and Tenovus Scotland (T20-69). CD holds research funding from JDRF, DiabetesUK and NIHR.
